# Lower Limb Inter-Joint Coordination and End-Point Control During Gait in Adolescents with Early Treated Unilateral Developmental Dysplasia of the Hip

**DOI:** 10.3390/bioengineering12080836

**Published:** 2025-07-31

**Authors:** Chu-Fen Chang, Tung-Wu Lu, Chia-Han Hu, Kuan-Wen Wu, Chien-Chung Kuo, Ting-Ming Wang

**Affiliations:** 1 Department of Biomedical Engineering, National Taiwan University, Taipei 10617, Taiwan; cfchang711@mail.tcu.edu.tw (C.-F.C.); d12528026@ntu.edu.tw (C.-H.H.); 2 Department of Physical Therapy, Tzu Chi University, Hualien 970374, Taiwan; 3 Health Science and Wellness Research Center, National Taiwan University, Taipei 10617, Taiwan; wukuanwen@gmail.com; 4 Department of Orthopaedic Surgery, School of Medicine, National Taiwan University, Taipei 10617, Taiwan; 5 Department of Orthopaedic Surgery, National Taiwan University Hospital, Taipei 10002, Taiwan; 6 Department of Orthopaedics, China Medical University Hospital, Taichung 40447, Taiwan; d4306kcc@gmail.com

**Keywords:** developmental dysplasia of the hip (DDH), Pemberton’s osteotomy, gait analysis, coordination, continuous relative phase, phase plot, variability

## Abstract

**Background**: Residual deficits after early treatment of developmental dysplasia of the hip (DDH) using osteotomy often led to asymmetrical gait deviations with increased repetitive rates of ground reaction force (GRF) in both hips, resulting in a higher risk of early osteoarthritis. This study investigated lower limb inter-joint coordination and swing foot control during level walking in adolescents with early-treated unilateral DDH. **Methods**: Eleven female adolescents treated early for DDH using Pemberton osteotomy were compared with 11 age-matched healthy controls. The joint angles and angular velocities of the hip, knee, and ankle were measured, and the corresponding phase angles and continuous relative phase (CRP) for hip–knee and knee–ankle coordination were obtained. The variability of inter-joint coordination was quantified using the deviation phase values obtained as the time-averaged standard deviations of the CRP curves over multiple trials. **Results**: The DDH group exhibited a flexed posture with increased variability in knee–ankle coordination of the affected limb throughout the gait cycle compared to the control group. In contrast, the unaffected limb compensated for the kinematic alterations of the affected limb with reduced peak angular velocities but increased knee–ankle CRP over double-limb support and trajectory variability over the swing phase. **Conclusions**: The identified changes in inter-joint coordination in adolescents with early treated DDH provide a plausible explanation for the previously reported increased GRF loading rates in the unaffected limb, a risk factor of premature OA.

## 1. Introduction

Developmental dysplasia of the hip (DDH) is characterized by a poorly developed acetabulum and displaced femoral head, often leading to hip joint instability and subluxation [1]. Undetected or untreated DDH can result in limb-length discrepancies, snapping sensation, and limited hip motion [2]. The hip incongruence and deformity also increase the joint contact pressures during walking [3], raising the risk of hip osteoarthritis (OA) [4]. In children with DDH, these changes in biomechanics lead to alterations in gait and movement control.

Early surgical correction for DDH is essential to achieve optimal functional outcomes and prevent the development of associated sequelae [5,6,7]. However, adolescents who underwent corrective surgery in infancy were reported to sustain repetitive loads of greater-than-normal rates on both hips around heel strike, increasing their risks of developing premature hip OA [8]. Similar findings have been observed in juveniles treated for unilateral DDH during toddlerhood, and the magnitudes of the loading rates in the lower limb joints during gait correlated with the morphology of the unaffected hip [8]. Thus, monitoring through gait analysis for changes in loading rates post-surgery is essential in identifying the residual sequelae of hip OA until growth maturity.

Human locomotion is a complex task that requires highly coordinated movements of body segments and continuous precise end-point motion control while negotiating environmental constraints to meet task demands. As a multi-segment system, the joint angles determine the position of the end-point of the lower limbs (i.e., foot segments) [9,10]; therefore, smooth and stable inter-joint coordination is essential for achieving precise control and maintaining dynamic stability during level walking [11,12]. During walking, a rapid increase in the ground reaction force (GRF) around the heel strike is linked to the impact of the foot when its velocity suddenly becomes zero [13]. Such high loading rates may initiate joint OA and accelerate damaged cartilage fibrillation, as has been observed in advanced OA [14]. Smooth endpoint contact with the ground around the heel strike can reduce the GRF loading rates, which can be achieved by proper control of the joint velocities of the lower limbs [15]. In contrast, compromised inter-joint coordination in limb control may lead to inaccurate endpoint contact, with a subsequent increase in GRF loading rates [16]. Residual deficits in the operated hip have been shown to lead to angular deviations or active compensatory changes in the lower-limb joints with high repetitive GRF loading rates during gait in children with DDH [17]. Our previous study also showed that adolescents with early-treated unilateral DDH exhibited asymmetrical gait patterns, characterized by anterior pelvic tilt, pelvic hiking on the affected side, pelvic rotation toward the unaffected side, and increased knee flexion and ankle dorsiflexion in the affected limb [13]. Thus, quantifying the coordinated movements between the lower limb joints in such patients may provide useful information to gain more insight into the effects of residual deficits on gait patterns in treated DDH hips.

The relationship between the angular positions and velocities of one joint relative to another is a common representation of inter-joint coordination involving the interplay between efferent motor control and afferent joint receptors [18]. Information on inter-joint coordination helps investigate how redundant degrees of freedom of the joints in the neuromusculoskeletal system are organized to achieve smooth, accurate, and efficient functional movement [19]. With the relative phase analysis method incorporating joint position and velocity information, the patterns and variabilities of inter-joint coordination during functional movement in various populations have been studied, including healthy young adults, older adults, and patients with diseases or injuries [11,20]. The variability observed in the relative phase plots from repeated trials served as an indicator of the stability of inter-joint coordination [20]. Generally, reduced variability is considered a lack of flexibility, while excessive variability may be associated with injury, injury risks, or conditions changing motor control patterns [18]. Considering the residual gait deviations in patients with an early reduction in unilateral DDH [13], different inter-joint coordination patterns with greater variability during level walking may be expected. More insight into lower limb inter-joint coordination may help explain the harmful loading rates around heel strike observed in patients with early reduction for unilateral DDH [17].

The current study aimed to determine the patterns and variability of inter-joint coordination in the lower extremities during gait in children treated for unilateral DDH during infancy, and to compare them with their typically developing peers. It was hypothesized that the DDH group would demonstrate altered bilateral hip–knee and knee–ankle coordination patterns during gait with greater variability than typically developing controls.

## 2. Materials and Methods

### 2.1. Participants

Eleven female adolescents with unilateral DDH treated using Pemberton osteotomy during infancy participated in the study (DDH group: age = 10.55 ± 0.99 years; age at surgery = 1.63 ± 0.47 years; postoperative duration = 8.64 ± 1.19 years; mass = 33.6 ± 8.3 kg; height = 140.82 ± 7.98 cm; leg length (LL) = affected limb = 73.27 ± 6.35 cm and unaffected limb = 72.27 ± 6.68 cm). A control group of eleven age-matched healthy peers was included for comparison (Control group: age = 10.99 ± 1.48 years; mass = 35.75 ± 7.74 kg; height = 143.52 ± 9.83 cm; LL = 76.12 ± 6.08 cm). Written consent was obtained from the participants after the Institutional Review Board approved the study and their parents or legal guardians provided informed consent. The DDH group was community ambulatory with no history of hip pain, infection, or other musculoskeletal disorders, and had not undergone any operation other than Pemberton’s osteotomy. According to the X-ray data, the frontal-plane acetabular coverage increased to within the normal range six months after surgery. The sample size in each group was determined through an a priori power analysis for a two-group independent *t*-test using G*POWER [21], based on pilot data of the deviation phase (DP) values for the hip–knee and knee–ankle continuous relative phase (CRP) curves, obtained from four participants in each group. The analysis indicated that a minimum of nine participants per group would be required to achieve a statistical power of 0.8 with a large effect size (Cohen’s d = 0.6) at a significance level of 0.05. Based on this estimation, 11 participants per group were considered appropriate for the current study.

### 2.2. Experimental Protocol

In the gait laboratory of a university hospital, every participant walked on an 8 m walkway at their preferred speed. The segmental motions of the pelvis–leg apparatus were measured using 28 retroreflective markers placed on standard anatomical landmark points commonly used in gait analysis [22,23]. Three-dimensional marker trajectories were measured using a motion capture system with seven infrared cameras (Vicon 512, Oxford Metrics Group, Oxford, UK) at 120 Hz and low-pass filtered using a fourth-order Butterworth filter with a cutoff frequency of 5 Hz before subsequent analysis, and the ground reaction forces (GRF) were measured using two force plates (AMTI, Advanced Mechanical Technology, Watertown, MA, USA) at 1080 Hz [13,24,25]. Each participant completed at least six successful trials, containing complete data for the entire gait cycle.

### 2.3. Data Analysis

Each segment of the pelvis–leg apparatus is represented as a rigid body. Each segment was fixed with an orthogonal coordinate system, with the positive *x*-axis directed anteriorly, positive *y*-axis superiorly, and positive *z*-axis directed right, following recommendations of the International Society of Biomechanics [26]. The angular displacement of a joint (joint angle) was calculated as the rotational movement of the distal segment relative to the proximal segment following a Cardanic rotation sequence of *z*-*x*-*y*. The angular velocities were calculated by fitting cubic splines to the time series of the angle and obtaining the first derivatives using the generalized cross-validation spline (GCVSPL) method [27]. Sign conventions of the angles and angular velocities follow the coordinate and Cardan angle definitions and are indicated in Figure 3 To reduce the negative effects of marker-skin movement artifacts, a global optimization method with joint constraints was used, which minimized the weighted sum of the squared distances of the model-experiment marker positions [17]. The peak values of the joint angles and angular velocities over the gait cycle, the values at ipsilateral and contralateral heel strike and toe off, and the subsequent ipsilateral heel strike were obtained for the statistical analysis. Standard temporospatial gait parameters, including speed, stride length, cadence, step length, and step width, were obtained. To assess the between-trial variability of the end-point trajectories, the standard deviations of the vertical and anterior–posterior components of the ensemble trajectories for the toe and heel markers were averaged over the swing phase. The stance phase was defined as the period from heel strike to toe off over the gait cycle.

**Figure 1:**
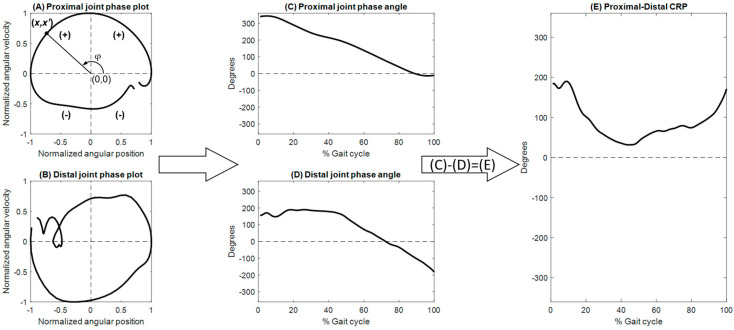
Schematic illustration of the analytical process for computing continuous relative phase (CRP). (**A**,**B**) depict the phase plots of the proximal and distal joints, respectively. (**C**,**D**) present the corresponding phase angles of the proximal and distal joints across the gait cycle, respectively. (**E**) shows the CRP profile obtained as the difference between the proximal and distal joint phase angles (**C**,**D**).

To generate the phase plots of the joints, the joint angular displacements were normalized to a range between −1 and 1, centered at zero. The angular velocity values were normalized based on the maximum absolute velocity over the gait cycle [11]. These normalization procedures are helpful in reducing the influence of different movement amplitudes and frequencies [28]. For each joint, a phase plot was generated by plotting the normalized angular velocities (*x*’) against the normalized angular displacements (x), and the angle from the positive *x*-axis to the ray from the origin to the point (*x*, *x*’) calculated using the 2-argument arctangent function yielded the phase angle (φ) [29]. Any phase angle discontinuity with magnitudes of π or greater was corrected to less than π by adding multiples of ±2π [20] (Figure 1).

**Figure 2:**
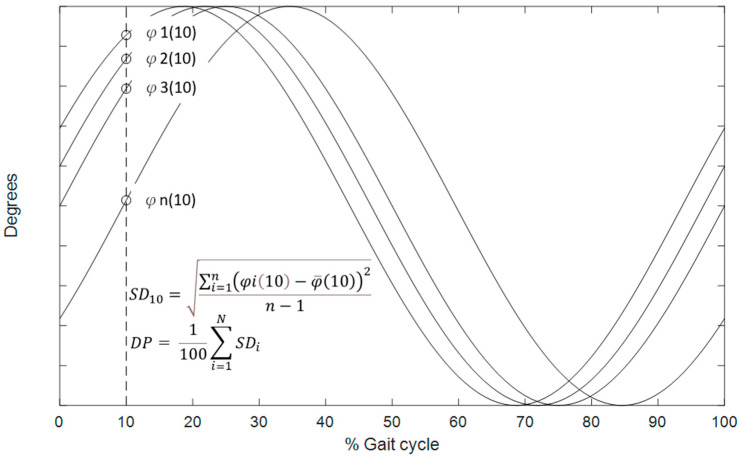
Schematic illustration of the computation of deviation phase (DP) values. At each time point of the gait cycle, the standard deviation (SDi) of continuous relative phase (CRP) values across multiple trials is calculated. The DP value is then obtained as the time-averaged standard deviation over the entire gait cycle, representing the variability of the CRP profiles.

The coordination between two adjacent joints was described by the CRP, which was calculated by subtracting the phase angle of the distal joint from that of the proximal joint [30]. In the current study, the coordination between the hip and knee was described by the hip–knee CRP ($\varphi_{hip-knee}$) and that between the knee and ankle by the knee–ankle CRP ($\varphi_{knee-ankle}$) (Figure 2). When the CRP was close to 0° or 360°, the two adjacent joints moved in a similar pattern or in-phase, whereas the CRP was close to 180°, representing an opposite or out-of-phase fashion. When the proximal joint leads the distal joint, the CRP value is positive, whereas a negative value indicates an opposite. The CRP curves of both limbs from the six walking trials were ensemble-averaged across all participants in each subject group to obtain general inter-joint coordination patterns. For each of the initial and terminal double-leg stance (DLS), single-limb support (SLS), and swing phases, phase-averaged CRP values for each inter-joint relationship were obtained. Another parameter, the coefficient of multiple correlations (CMC) [20] was used to quantify the similarity of CRP patterns between the Control and DDH groups. A CMC value approaching unity indicates that the patterns were congruent. Root-mean-squared differences (RMSD) were used to quantify between-group differences in the ensemble-averaged CRP curves for each phase. While CMC identified temporal differences in phase shifts, RMSD characterized deviations in the relative phase pattern magnitudes and fluctuations. Therefore, a high CMC with a low RMSD denotes a similar pattern and magnitude between the two given curves. The CMC values were interpreted as follows: 0.65–0.75, moderate, 0.75–0.85 indicates good, 0.85–0.95 is very good, and 0.95–1 is excellence level [31]. The variability of inter-joint coordination was quantified by the DP values obtained as the time-averaged standard deviations of the CRP curves for each gait phase over multiple trials [28] (Figure 2). A low DP value indicates a less variable and more stable inter-joint coordination relationship.

### 2.4. Statistical Analysis

The Shapiro–Wilk test was used to determine the normality of all calculated variables. Independent *t*-tests were used to compare differences between the groups for normally distributed data, whereas paired *t*-tests were used to identify differences between the affected and unaffected limbs in the DDH group. For non-normally distributed variables, between-group differences were assessed using the Mann–Whitney U-test, whereas differences between the affected and unaffected limbs of the DDH group were detected using the Wilcoxon signed-rank test. A significance level of 0.05 was set for all tests.

## 3. Results

**Figure 3:**
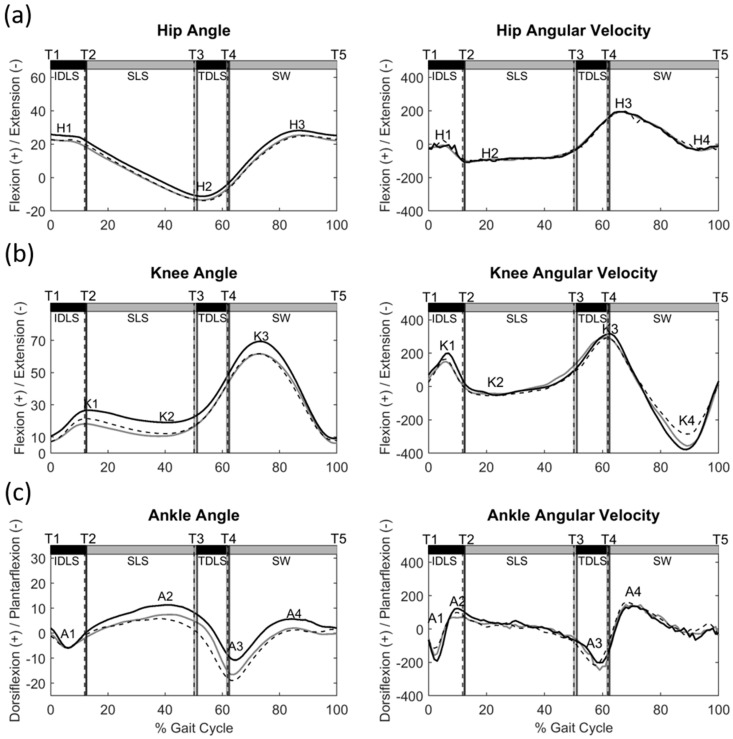
Ensemble−averaged angles (°) and angular velocities (°/s) of (**a**) the hip, (**b**) knee and (**c**) ankle of the affected limb (black, solid line) and unaffected limb (black, dotted line) in the DDH group and Control group (grey, solid line) during level walking. T1: Heel strike; T2: Contralateral toe off; T3: Contralateral heel strike; T4: Toe off; T5: Subsequent heel strike; H1−H4: Peaks and troughs in the hip curves; K1−K4: Peaks and troughs in the knee curves; A1−A4: peaks and troughs in the ankle curve.

Compared with the control group, individuals with DDH showed a significant increase in step width, but without significant differences in the other spatiotemporal parameters (Table 1). The DDH group also demonstrated a significant increase in deviation values for the end-point trajectories (both toe and heel) over the swing phase in the unaffected limb compared to the control group (Table 1). Significant differences in the joint angles were found between the affected limb and control, and between the affected and unaffected limbs, while differences in the joint angular velocities were found between the unaffected limb and control (Figure 3 and Supplementary Table S1). Over the entire gait cycle, the DDH group exhibited a more flexed posture in the affected limb with greater knee flexion and ankle dorsiflexion than the control group but without significant differences in joint angular velocities. In contrast, the unaffected limb showed significantly higher hip flexion peak velocity and ankle plantarflexion peak velocity, but lower knee joint angular velocities around toe off, without significant differences in the joint angles. The affected limb showed greater knee flexion and ankle dorsiflexion but a smaller hip extension than the unaffected limb during the entire gait cycle. No significant differences between the limbs were found in the angular velocities of any joint.

**Figure 4:**
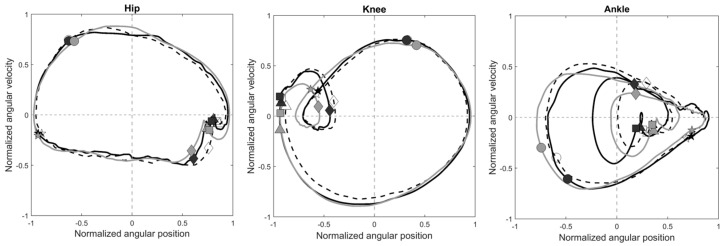
Ensemble−averaged phase plots of the hip, the knee and the ankle of the affected limb (black, solid line) and unaffected limb (black, dotted line) in the DDH group and the Control group (grey, solid line) during level walking. Square markers: Heel strike; Diamond markers: Contralateral toe off; Pentagram markers: Contralateral heel strike; Circular markers: Toe off; Triangular markers: Subsequent heel strike.

**Figure 5:**
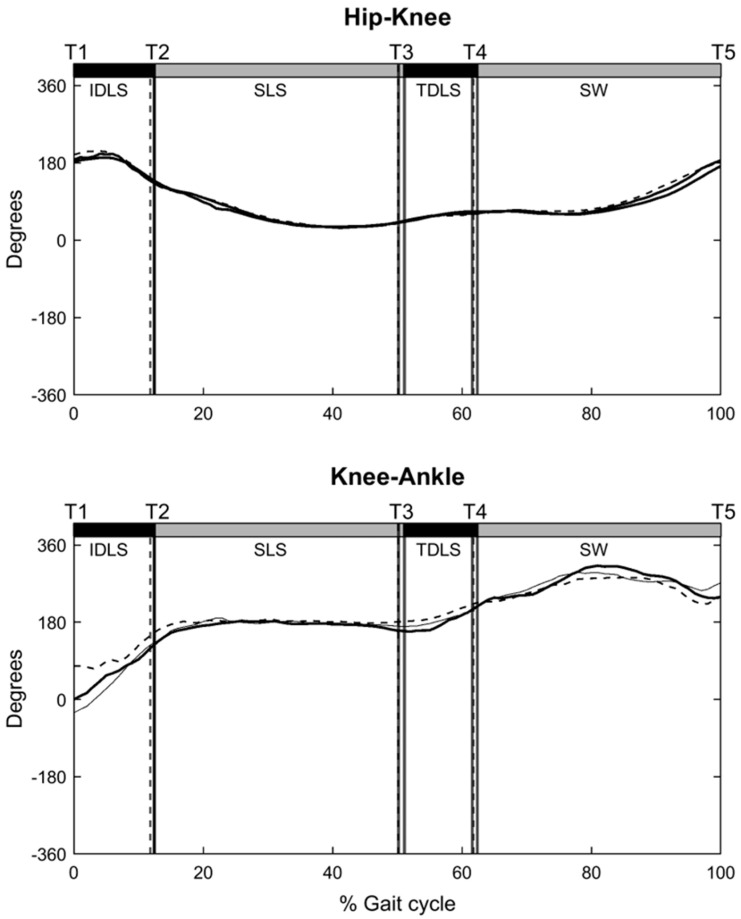
Ensemble−averaged CRP curves of the hip−knee and knee−ankle coordination of the affected limb (black, solid line) and unaffected limb (black, dotted line) in the DDH group and the Control group (grey, solid line) during gait.

Both groups showed nearly closed limit circles for the phase trajectories of the hip, knee, and ankle; however, the phase plots for the ankle exhibited different trajectories (Figure 4). The differences between the groups in each phase trajectory were visible during DLS (Figure 5).

Both groups displayed similar patterns of inter-joint coordination in the hip–knee and knee–ankle CRP curves (Figure 5). For the hip–knee CRP patterns over the gait phases, good to excellent correlations (CMC: 0.79 to 0.94) with the control group were found for both the affected and unaffected limbs of the DDH group (Table 2). No significant between-group or between-limb differences in the phase-averaged CRP values for the DDH group were found in the hip–knee CRP curves (*p* > 0.05; Table 2), with between-group RMSD values ranging from 2.52° to 13.53° for both the affected and unaffected limbs (Table 2). No significant between-group differences were found in DP values for any gait phase, regardless of the affected or unaffected limb (*p* > 0.05; Table 3).

For the knee–ankle CRP patterns over the gait phases, the affected limb showed good to excellent correlation with the control group (CMC: 0.75 to 0.85), while the unaffected limb showed almost no correlation with the control (Table 2). No significant between-group or between-limb differences were found in the phase-averaged CRP values for most gait phases (*p* > 0.05), except for the initial and terminal DLS of the unaffected limb, which showed significantly greater phase-averaged CRP values than the control (*p* < 0.05; Table 2). The RMSD values for the affected and unaffected limbs ranged from 5.46° to 15.69° over most gait phases, except for the initial and terminal DLS of the unaffected limb, which had RMSD values of 20.94° and 40.93°, respectively (Table 3). For inter-joint coordination variabilities, significantly greater DP values of the knee–ankle CRP in the affected limb were found during the stance phase of the DDH group (i.e., DLS and SLS) than in the controls (Table 3).

## 4. Discussion

The DDH group adopted a more flexed posture in the affected limb throughout the gait cycle with significantly increased knee flexion and ankle dorsiflexion compared to the Control, but with no significant differences in joint angular velocities (Table 2). The observed more flexed posture of the affected limb may be related to residual deficits in muscle strength, such as hip flexors and abductors, and knee extensors in the affected limb, which are often observed in patients treated with DDH [13]. While the observed angular displacement and velocity changes corresponded with altered phase trajectories of the knee and ankle (Figure 5), the inter-joint coordination patterns in the affected limb remained comparable to those of healthy controls, as suggested by the good to excellent correlations and low RMSD of the CRP curves (Table 3). In contrast, although the unaffected limb showed similar joint angles to the controls, differences in joint angular velocities around initial and terminal DLS were observed (Table 2), which were associated with variations in the phase trajectories of the knee and ankle (Figure 5) and lower correlations with the knee–ankle inter-joint coordination patterns of the healthy controls. From the current results, it appears that the unaffected limb compensated for the changes in the affected limb by altering the joint angular velocities, which are more likely controlled by normal muscle strength, and thus knee–ankle coordination to maintain close to normal inter-joint coordination patterns in the affected limb.

While the affected limb showed inter-joint coordination patterns similar to those of the healthy control, the knee–ankle coordination during the stance phase was less stable than that of the control group, with DP values greater than those of the control. Excessive variability may indicate a less stable movement pattern [11,18]. The greater variability of the knee–ankle coordination in the affected limb during stance corresponded to the greater variability of the swing foot and toe trajectories of the unaffected limb during the swing phase and shortly before heel strike. Precision control of the endpoint of the lower limb throughout the swing phase is essential for smooth heel contact to reduce GRF loading rates [9,16,32]. Previous studies have found that adolescents treated for DDH show significantly increased loading rates in both the affected and unaffected limbs [8]. It has been suggested that impaired control over the swing limb after osteotomy may increase the loading rates of the GRF, extending to the lower limb joints [33]. The current findings provide a possible explanation for increased GRF loading rates observed in the unaffected limb. That is, the less stable control of the knee–ankle inter-joint coordination in the affected limb during stance led to greater variability in the trajectories of the swing foot and endpoints and the subsequent impact on the ground, and thus increased GRF loading rates, a factor closely related to the development of OA [8].

Only female adolescents with early treated unilateral DDH were included in the current study because females are 5–9 times more likely to be affected than males [34], making it feasible to recruit a clinically homogeneous sample. Although sex differences in motor control strategies have been reported, particularly in adults, this study focused on a typical adolescent female cohort without aiming to examine sex-related differences in movement control. Further research may be needed to determine whether male patients have comparable inter-joint control strategies. Future investigations may also include patients with bilateral DDH. Functional tasks that demand more joint motion, such as stair walking and sit-to-stand movements, may be needed to provide deeper insights into the mechanical functions of DDH. Future investigations may benefit from comparing patient-specific joint angles with the established normative range of motion data to better characterize active compensatory strategies in both the affected and unaffected limbs. Including the trunk segment in inter-joint coordination in other planes may be necessary for pathological conditions that involve whole-body movement beyond the sagittal plane.

## 5. Conclusions

In addition to significant changes in the individual joint kinematics, the adolescents with early treated DDH also changed the way the motions of the lower limb joints are coordinated during level walking and displayed asymmetrical inter-joint coordination patterns between limbs. Compared to the Control, the DDH group showed a more flexed posture with increased variability in knee–ankle CRP curves in the affected limb throughout the gait cycle. It appears that the unaffected limb compensated for the kinematic alterations of the affected limb with decreased peak joint angular velocities but increased the knee–ankle CRP mean values over double-limb support and trajectory variability over the swing phase. The observed greater variability in the knee–ankle inter-joint coordination of the affected limb during stance and the trajectories of the end-points of the unaffected swing foot provide a possible explanation for the increased GRF loading rates in the unaffected limb, a risk factor for the initiation of OA at the lower limb joints.

## Figures and Tables

**Table 1:** Means (standard deviations) of the spatiotemporal parameters and the deviation values of the heel/toe end-point trajectories in the DDH and Control groups.

	Control Group	DDH Group		Pg		Ps
		Affected	Unaffected	Affected	Unaffected	
Gait speed (m/s)	1.22 (0.24)	1.13 (0.13)	1.10 (0.23)	0.306	0.314	0.819
Stride length (mm)	112.31 (9.93)	110.91 (8.52)	111.02 (7.00)	0.730	0.741	0.904
Cadence (1/s)	118.72 (12.82)	111.10 (10.94)	113.63 (12.40)	0.162	0.365	0.353
Step length (mm)	579.93 (49.23)	579.5 (39.75)	560.6 (31.01)	0.983	0.300	0.054
Step width (mm)	832.52 (268.51)	1070.30 (389.52)		0.001 *		
End-points (during the swing phase): Heel						
Anterior-posterior component (mm)	22.69 (9.12)	35.83 (24.37)	36.31 (17.71)	0.110	0.035 *	0.959
Vertical component (mm)	11.99 (5.88)	19.98 (15.19)	20.65 (10.78)	0.119	0.030 *	0.906
End-points (during the swing phase): Toe						
Anterior-posterior component (mm)	28.07 (10.65)	40.65 (31.97)	39.38 (15.68)	0.230	0.042 *	0.907
Vertical component (mm)	5.35 (0.85)	8.47 (5.05)	9.80 (2.74)	0.069	0.001 *	0.451

Pg: *p*-values for between-group comparisons using U-test; Ps: *p*-values for between-limb comparisons in the DDH group using Wilcoxon signed ranks test; *: Significant difference (*p* < 0.05).

**Table 2:** Means (standard deviations) of phase-averaged CRP of the hip–knee and knee–ankle coordination and coefficient of multiple correlation (CMC) values and the RMS differences (RMSD) of the CRP curves between DDH and Control groups.

Phase	CRP	Control Group	DDH Group		Pg		Ps	CMC		RMSD	
			Affected	Unaffected	Affected	Unaffected		Affected	Unaffected	Affected	Unaffected
Initial DLS	Hip–Knee	177.99 (13.08)	180.75 (16.62)	187.01 (11.07)	0.670	0.096	0.311	0.94	0.83	6.05	11.57
	Knee–Ankle	62.21 (77.97)	43.62 (42.29)	97.66 (57.41)	0.495	0.023 *	0.210	0.85	0.31	15.32	40.93
SLS	Hip–Knee	63.45 (9.87)	59.67 (9.04)	63.48 (8.88)	0.360	0.994	0.330	0.97	0.99	4.97	2.52
	Knee–Ankle	172.30 (9.77)	174.12 (7.44)	179.69 (8.27)	0.629	0.700	0.112	0.75	0.02	5.46	9.46
Terminal DLS	Hip–Knee	56.75 (11.72)	55.18 (10.54)	52.68 (8.69)	0.743	0.366	0.552	0.91	0.79	2.74	4.26
	Knee–Ankle	178.24 (18.43)	184.52 (21.92)	196.39 (16.97)	0.475	0.026 *	0.171	0.76	0.09	10.01	20.94
Swing	Hip–Knee	89.18 (9.03)	91.93 (6.54)	96.38 (13.86)	0.424	0.164	0.346	0.94	0.86	8.99	13.53
	Knee–Ankle	277.99 (35.92)	268.57 (20.11)	257.21 (22.49)	0.456	0.119	0.226	0.75	0.71	14.19	15.69

SLS: Single limb support; DLS: Double limb support; Pg: *p*-values for between-group comparisons using U-test; Ps: *p*-values for between-limb comparisons in the DDH group using Wilcoxon signed ranks test; *: Significant difference (*p* < 0.05).

**Table 3:** Means (standard deviation) of the DP values for CRP curves of the DDH and Control groups.

Phase	CRP	Control Group	DDH Group		Pg		Ps
			Affected	Unaffected	Affected	Unaffected	
Initial DLS	Hip–Knee	16.80 (8.33)	26.00 (9.92)	22.22 (8.42)	0.360	0.163	0.369
	Knee–Ankle	8.81 (2.92)	11.72 (5.51)	10.31 (3.78)	0.040 *	0.156	0.514
SLS	Hip–Knee	8.49 (4.02)	6.59 (3.19)	6.63 (3.04)	0.254	0.256	0.976
	Knee–Ankle	7.86 (2.87)	11.69 (4.41)	8.97 (4.30)	0.032 *	0.507	0.176
Terminal DLS	Hip–Knee	66.44 (34.64)	62.77 (27.48)	55.21 (27.93)	0.796	0.434	0.549
	Knee–Ankle	12.49 (4.06)	15.49 (10.08)	14.44 (5.97)	0.039 *	0.404	0.777
Swing	Hip–Knee	13.77 (6.04)	16.02 (7.54)	18.05 (6.37)	0.469	0.138	0.522
	Knee–Ankle	22.46 (9.74)	29.02 (14.79)	26.31 (15.43)	0.255	0.513	0.692

SLS: Single limb support; DLS: Double limb support; Pg: *p*-values for between-group comparisons using U-test; Ps: *p*-values for between-limb comparisons in the DDH group using Wilcoxon signed ranks test; *: Significant difference (*p* < 0.05).

## Data Availability

The datasets used and/or analyzed during the current study are available from the corresponding author upon reasonable request.

## Supplementary Materials

The following supporting information can be downloaded at: https://www.mdpi.com/article/10.3390/bioengineering12080836/s1, Table S1: Means (standard deviations) of the angles and angular velocities at the hip, knee and ankle joints in the DDH and Control groups.
