# Supplementary material for: Lower Limb Inter-Joint Coordination and End-Point Control During Gait in Adolescents with Early Treated Unilateral Developmental Dysplasia of the Hip
Source: Bioengineering (Basel). 2025 Jul 31;12(8):836. doi: 10.3390/bioengineering12080836 (PMC12383746; doi:10.3390/bioengineering12080836)
Supplement: Supplementary file 1 [file bioengineering-12-00836-s001.zip › bioengineering-3715007-supplementary.pdf]

SUPPLEMENTAL MATERIALS

Supplementary Table S1.— *Means (standard deviations) of the angles and angular velocities at the hip, knee and ankle joints in the DDH and Control groups.*

	Control group	DDH group		Pg		Ps
		Affected	Unaffected	Affected	Unaffected	
**Joint angle (degree)**						
**Hip: Flexion (+) / Extension (-)**						
T1	22.79 (4.66)	25.90 (3.67)	22.55 (4.56)	0.136	0.912	0.274
H1	22.94 (4.70)	26.36 (3.79)	25.43 (5.07)	0.109	0.295	0.672
T2	17.96 (5.93)	21.43 (3.23)	20.58 (5.97)	0.142	0.363	0.686
T3	-12.95 (3.39)	-10.97 (5.67)	-13.23 (3.30)	0.382	0.864	0.164
H2	-13.69 (3.96)	-11.81 (5.23)	-14.49 (3.67)	0.404	0.659	0.150
T4	-6.32 (5.86)	-2.76 (7.58)	-7.68 (3.10)	0.581	0.546	0.049*
H3	26.24 (4.01)	28.46 (4.00)	23.63 (3.86)	0.255	0.179	0.052
T5	22.10 (4.34)	25.16 (2.67)	23.61 (4.44)	0.090	0.477	0.393
**Knee: Flexion (+) / Extension (-)**						
T1	7.04 (3.05)	10.51 (6.30)	7.20 (4.77)	0.156	0.930	0.203
T2	18.25 (5.23)	26.51 (6.71)	21.44 (6.81)	0.010*	0.283	0.128
K1	18.54 (5.11)	27.19 (6.87)	21.89 (6.94)	0.008*	0.261	0.063
K2	10.14 (3.45)	17.35 (6.61)	11.52 (7.05)	0.010*	0.605	0.027*
T3	17.04 (5.05)	23.72 (6.70)	16.83 (7.61)	0.030*	0.945	0.031*
T4	43.31 (8.14)	51.89 (8.55)	44.52 (5.54)	0.045*	0.718	0.102
K3	62.28 (7.18)	70.15 (7.92)	63.31 (6.79)	0.042*	0.758	0.138
T5	6.19 (4.23)	9.26 (5.83)	8.02 (7.08)	0.220	0.517	0.640
**Ankle: Plantar flexion (+) / Dorsi-flexion (-)**						
T1	-0.74 (3.10)	2.04 (1.95)	-1.15 (4.31)	0.037*	0.821	0.085*
A1	-6.24 (4.29)	-6.08 (3.38)	-6.16 (3.09)	0.929	0.963	0.955
T2	-1.68 (2.30)	0.75 (3.73)	-1.13 (3.32)	0.115	0.686	0.234
A2	7.44 (3.59)	11.91 (2.99)	6.74 (4.84)	0.009*	0.725	0.011*
T3	2.75 (5.91)	7.89 (3.01)	1.25 (4.82)	0.034*	0.562	0.006*
T4	-16.58 (6.08)	-10.49 (5.25)	-18.46 (7.70)	0.037*	0.573	0.033*
A3	-17.05 (7.91)	-11.90 (5.88)	-19.96 (8.21)	0.129	0.442	0.041*
A4	2.78 (1.98)	6.79 (3.37)	3.00 (6.57)	0.005*	0.920	0.168
T5	-0.41 (3.00)	2.03 (4.02)	1.33 (4.45)	0.163	0.344	0.713
**Joint angular velocity (degree/s)**						
**Hip: Flexion (+) / Extension (-)**						
T1	-22.97 (26.89)	-26.38 (44.23)	-20.84 (25.54)	0.846	0.865	0.753
H1	-2.79 (25.65)	8.31 (29.00)	31.23 (31.28)	0.402	0.023*	0.098
T2	-98.14 (21.13)	-96.47 (32.43)	-88.39 (29.84)	0.899	0.409	0.512
H2	-117.22 (22.88)	-135.11 (26.12)	-129.43 (24.81)	0.142	0.294	0.629
T3	-31.69 (25.70)	-30.06 (35.70)	-33.13 (28.48)	0.913	0.875	0.682
T4	159.38 (16.09)	163.40 (24.33)	166.16 (41.97)	0.684	0.511	0.668
H3	202.11 (13.62)	210.75 (41.36)	206.22 (56.06)	0.560	0.834	0.871
H4	-50.91 (35.09)	-49.57 (22.49)	-50.43 (42.03)	0.924	0.979	0.961

T5	-17.43 (29.21)	-10.92 (37.65)	-7.53 (25.65)	0.687	0.404	0.573
Knee: Flexion (+) / Extension (-)						
T1	41.72 (49.32)	68.77 (71.27)	24.78 (65.68)	0.363	0.545	0.259
K1	155.21 (74.16)	210.07 (46.94)	181.25 (82.26)	0.079	0.491	0.358
T2	0.37 (33.64)	12.67 (56.46)	4.39 (47.26)	0.582	0.838	0.749
K2	-61.00 (34.14)	-53.75 (47.34)	-78.50 (40.59)	0.714	0.337	0.196
T3	132.07 (53.39)	117.26 (82.42)	81.11 (45.65)	0.657	0.045*	0.218
T4	300.09 (36.29)	319.07 (44.09)	303.74 (57.08)	0.334	0.873	0.594
K3	310.75 (30.86)	331.08 (50.14)	313.21 (56.02)	0.316	0.910	0.562
K4	-366.37 (67.48)	-404.98 (51.49)	-327.36 (118.33)	0.191	0.403	0.124
T5	15.20 (95.94)	32.59 (77.70)	8.11 (87.60)	0.678	0.872	0.450
Ankle: Plantar flexion (+) / Dorsi-flexion (-)						
T1	-51.68 (43.92)	-63.97 (41.75)	-69.53 (50.21)	0.551	0.434	0.736
A1	-177.96 (73.08)	-201.94 (103.90)	-145.99 (52.17)	0.579	0.301	0.192
A2	107.48 (40.03)	131.74 (37.23)	131.36 (53.44)	0.202	0.299	0.982
T2	69.25 (23.43)	86.21 (26.16)	97.36 (58.75)	0.167	0.201	0.443
T3	-48.90 (25.14)	-62.12 (35.56)	-80.99 (58.52)	0.376	0.150	0.392
A3	-293.33 (48.16)	-258.01 (72.89)	-243.05 (47.15)	0.243	0.040*	0.465
T4	-162.84 (103.26)	-88.90 (144.72)	-81.85 (98.34)	0.230	0.108	0.918
A4	194.76 (51.30)	173.63 (53.79)	198.47 (45.13)	0.406	0.873	0.192
T5	-39.40 (43.74)	-22.82 (90.36)	-59.01 (74.39)	0.627	0.505	0.084

T1: Heel-strike; T2: Contralateral toe-off; T3: Contralateral heel-strike; T4: Toe-off; T5: Subsequent heel-strike; H1-H4: Peaks and troughs in the hip curves; K1-K4: Peaks and troughs in the knee curves; A1-A4: Peaks and troughs in the ankle curves; Pg: p-values for between-group comparisons using U-test; Ps: p-values for between-limb comparisons in the DDH group using Wilcoxon signed ranks test; *: Significant difference ($p<0.05$)
